# Diagnostic challenges of histidine-rich protein 2-based rapid diagnostic tests due to *pfhrp2* and *pfhrp3* gene deletions in asymptomatic malaria in Tanzania

**DOI:** 10.1186/s40249-025-01397-3

**Published:** 2026-03-09

**Authors:** Ernest Mazigo, Hojong Jun, Wang-Jong Lee, Johnsy Mary Louis, Fadhila Fitriana, Jadidan Hada Syahada, Fauzi Muh, Wanjoo Chun, Won Sun Park, Se Jin Lee, Sunghun Na, Eun-Taek Han, Feng Lu, Winifrida Kidima, Jin-Hee Han

**Affiliations:** 1 Department of Medical Environmental Biology and Tropical Medicine, School of Medicine, Kangwon National University, Chuncheon, Gangwon Republic of Korea; 2 Department of Parasitic Diseases, National Institute for Medical Research, Dar es Salaam, Tanzania; 3 Department of Epidemiology and Tropical Diseases, Faculty of Public Health, Universitas Diponegoro, Semarang, Indonesia; 4 Department of Pharmacology, School of Medicine, Kangwon National University, Chuncheon, Republic of Korea; 5 Department of Physiology, School of Medicine, Kangwon National University, Chuncheon, Republic of Korea; 6 Department of Obstetrics and Gynecology, Kangwon National University Hospital, Chuncheon, Republic of Korea; 7 Department of Pathogen Biology and Immunology, School of Medicine, Yangzhou University, Yangzhou, China; 8 Department of Zoology, College of Natural and Applied Sciences, University of Dar es Salaam, Dar es Salaam, Tanzania; 9 Institute of Medical Sciences, Kangwon National University, Chuncheon, Republic of Korea

**Keywords:** *Plasmodium falciparum*, *pfhrp2*, *pfhrp3*, Rapid diagnostic test, Asymptomatic malaria, Tanzania, Malaria elimination

## Abstract

**Background:**

Histidine-rich protein 2 (HRP2)-based rapid diagnostic tests (RDTs) are critical for malaria diagnosis in Africa, particularly in resource-limited settings. However, the spread of *Plasmodium falciparum* parasites with *pfhrp2* and *pfhrp3* gene deletions challenges their effectiveness, raising concerns in affected areas. Therefore, this study aimed to assess the prevalence of *pfhrp2* and *pfhrp3* gene deletions and evaluate the diagnostic performance of HRP2-based RDTs in detecting asymptomatic malaria infections in Tanzania.

**Method:**

This cross-sectional community survey study aimed to determine the prevalence of *pfhrp2/3* gene deletions from asymptomatic malaria population in Tanzania. Moreover, the study intended to assess the performance of HRP2-based RDT and light microscopy (LM) in detecting asymptomatic malaria infections. The survey was conducted from December 2022 to July 2023 in twelve villages (8 villages from high malaria transmission areas and 4 in low endemicity). Diagnosis was done by RDT, LM and quantitative polymerase chain reaction (qPCR). A multiplex qPCR was employed on *P. falciparum* mono-infection isolates to assess the prevalence of *pfhrp2/3* gene deletions. Unpaired *t*-tests and one-way ANOVA were performed to evaluate associations between log_10_-transformed parasitaemia levels and gene deletion profiles.

**Results:**

Among 3489 participants, RDT detected 710 (77.6%) of 915 qPCR-positive cases, compared to 492 (53.8%) by LM. Compared with qPCR, RDT produced 143 (5.6%) false positives and 205 (22.4%) false negatives, whereas LM had 60 (2.3%) false positives and 423 (46.2%) false negatives. Overall accuracy was similar for RDT (90.0%) and LM (86.2%), with higher sensitivity for RDT. Agreement with qPCR in asymptomatic cases was substantial for RDT (κ = 0.736), whereas it was moderate for LM (κ = 0.590). Multiplex qPCR revealed *pfhrp2* or *pfhrp3* deletions in 93 (11.8%) of 787 samples, including 19 (2.4%) dual-deletion isolates, all of which were RDT negative. Deletions were more frequent in high-transmission villages (76 cases, 9.7%) than in low-transmission villages (17 cases, 2.2%).

**Conclusion:**

The findings support the continued effectiveness of HRP2-based RDTs in detecting asymptomatic *P. falciparum* infections in Tanzania, despite the presence of some *pfhrp2/3* gene deletions. However, the potential increase in deletion prevalence and the limitations of cross-sectional data highlight the need for ongoing molecular surveillance. Collectively, these findings provide a critical foundation for sustained surveillance and for clarifying the epidemiological significance of asymptomatic malaria in Tanzania.

**Graphical Abstract:**

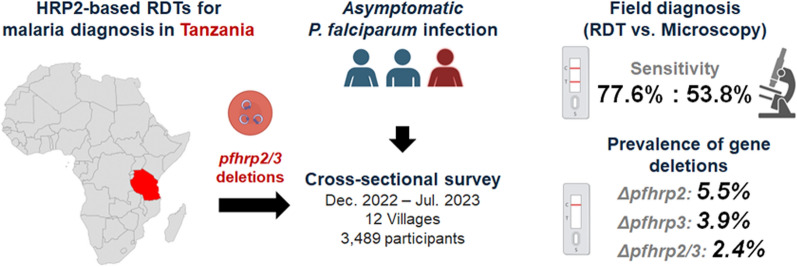

**Supplementary Information:**

The online version contains supplementary material available at 10.1186/s40249-025-01397-3.

## Background

Malaria is still a public health problem globally, particularly in sub-Saharan Africa (SSA) countries, which account for 94% of the global malaria cases and 95% of deaths, respectively [1]. Evidence shows a resurgence of malaria in areas where the disease has significantly declined [2, 3]. This observation calls for assessment of the effectiveness of malaria control strategies in use [2]. Light microscopy (LM), a standard assay for malaria diagnosis, faces several operational challenges, including limited expertise, power constraints, and time-consuming procedures, leading to delays in reporting results [4, 5]. These challenges necessitate the use of rapid diagnostic tests (RDTs) particularly in rural areas [6]. RDTs typically yield results within 15–20 min, enabling faster clinical decisions compared to LM. Moreover, modern RDTs are sensitive enough to detect low levels of parasitaemia, sometimes even below the threshold detectable by LM [7].

Of the five *Plasmodium* parasites that are infective to humans, *P. falciparum* accounts for more than 95% of malaria infections in SSA [8]. Based on this observation, the majority of RDTs distributed in SSA are primarily designed using histidine-rich protein 2 (HRP2) as an antigen target to detect *P. falciparum* parasites. The HRP2 is a water-soluble protein with a molecular size of 60–105 kDa which is secreted by asexual trophozoite stages of *P. falciparum* parasites [9, 10]. HRP2 is located on chromosome 8 of the parasite and is secreted in abundance, and circulates in peripheral blood even when the parasites are sequestered in the microvasculature of organs [10, 11]. This makes the protein an ideal target for diagnosis of the *P. falciparum* parasites. The HRP2 is known to have 85%–90% similarity of nucleotide sequences with the HRP3. This homology renders a cross-reactivity with monoclonal antibodies in HRP2-based RDTs especially in higher parasite densities [12]. Thus, assessing the prevalence of both proteins is crucial as only parasites with dual deletions can be missed by the HRP2-based RDTs [13, 14].

In Tanzania, the National Malaria Control Program (NMCP) initiated the use of RDT for the diagnosis of malaria in 2009. Individuals suspected of malaria cases were required to be confirmed by the HRP2-based RDT before treatments. By 2014, the coverage of RDT across public and private health facilities in Tanzania was about 90%, and RDT is currently the first-line diagnostic assay for the screening of symptomatic malaria in all health facilities [15]. Five different brands of RDTs were approved by the Tanzania Medical Device Authority (TMDA), and in 2015, each outlet in mainland Tanzania sold approximately 40 RDT units per week, with more than 6000 outlets in total [16–18]. Nevertheless, evidence indicates that *pfhrp2/3* gene deletions are present in *P. falciparum* parasites in several countries, including Tanzania [19–21]. Parasites with such deletions are missed by HRP2-based RDTs resulting in false-negative results [20, 22]. Nonetheless, most studies on *pfhrp2/3* gene deletions focus on clinical malaria infections and less consideration is given to asymptomatic individuals with malaria.

Reports on the prevalence of individuals with malaria parasites without symptoms are increasing, particularly in areas with high malaria transmission including Tanzania [23–25]. Asymptomatic malaria carriers are infectious and serve as silent precursors to symptomatic infections [23, 25]. Thus, if these silent infections involve parasites with *pfhrp2/3* gene deletions, they may also be missed by the HRP2-based RDTs and continue circulating undetected within the population. This selection pressure can increase the frequency of these RDT-evading strains, particularly in regions where RDTs are the primary diagnostic tool, potentially complicating malaria control and elimination efforts.

It is against this background that the present study aimed to assess the prevalence of *pfhrp2/3* gene deletions in asymptomatic malaria carriers across villages with varying endemicity in Tanzania. A previous survey of the same cohort reported asymptomatic malaria prevalence using RDT and LM [25]. The current study extends these findings by integrating molecular diagnostics to evaluate *pfhrp2/3* deletions, assess RDT performance in asymptomatic infections, and examine associations with demographic factors. By focusing on asymptomatic carriers, this study provides evidence on the contribution of asymptomatic infections to malaria transmission and the potential impact of *pfhrp2/3* deletions on RDT-based surveillance and control strategies.

## Methods

### Geographic coverage and study design

Blood samples were obtained from a cross-sectional community-based study. The study was undertaken from December 2022 to July 2023 and involved 12 villages with different levels of malaria endemicity in Tanzania [25]. Of the twelve villages, eight were from high-malaria transmission hotspots and four were from low-transmission settings. Through multi-stage sampling, two regions (Kigoma and Geita) were purposively selected to represent areas with high malaria cases, and one region (Arusha) was selected to represent areas with low prevalence (Supplementary Table S1). Thereafter, two districts from each region were randomly selected, followed by the selection of two villages per district. The two districts and two villages from Geita region were Nyang’hwale district (Nyangalamila and Kayenze) and Chato district (Ihanga and Rwantaba). From Kigoma region, the districts were Kibondo district (Kumuhasha and Bunyambo) and Kasulu district (Nyamnyusi and Mugombe) while from Arusha, Meru district (Maji ya Chai and Ngurudoto) and Arusha District Council (Bwawani and Themi ya Simba) (Table 1).

**Table 1:** Distribution of collected samples in study sites and isolates selected for detection of *pfhrp2* and *pfhrp3* gene deletions

	qPCR results, *n* (%)	RDT results, *n* (%)	*P. falciparum* mono-infections selected for *hrp2/3* detection, *n* (%^a^)				
Site (*n*)	qPCR +	Pan +	Selected isolates	qPCR +	RDT + & qPCR +	LM + & qPCR +	qPCR +, LM + & RDT +
Nyangalamila (276)	102 (37.0)	8 (2.9)	94 (34.1)	22 (23.4)	25 (26.6)	3 (3.2)	44 (46.8)
Kayenze (327)	81 (24.8)	4 (1.2)	77 (23.5)	7 (9.1)	11 (14.3)	6 (7.8)	53 (68.8)
Rwantaba (278)	116 (41.7)	30 (10.8)	86 (30.9)	6 (7.0)	39 (45.3)	6 (7.0)	35 (40.7)
Ihanga (339)	126 (37.2)	27 (8.0)	99 (29.2)	6 (6.1)	41 (41.4)	3 (3.0)	49 (49.5)
GEITA (1220)	425 (34.8)	69 (5.7)	356 (29.2)	41 (11.5)	116 (35.6)	18 (5.1)	181 (50.8)
Kumuhasha (293)	94 (32.1)	21 (7.2)	73 (24.9)	9 (12.3)	29 (39.7)	4 (5.5)	31 (42.5)
Bunyambo (303)	113 (37.3)	13 (4.3)	100 (33.0)	30 (30.0)	31 (31.0)	5 (5.0)	34 (34.0)
Nyamnyusi (259)	124 (47.9)	9 (3.5)	115 (44.4)	35 (30.4)	11 (9.6)	4 (3.5)	65 (56.5)
Mugombe (290)	112 (38.6)	11 (3.8)	101 (34.8)	25 (24.8)	11 (10.9)	6 (5.9)	59 (58.4)
KIGOMA (1145)	443 (38.7)	54 (4.7)	389 (34.0)	99 (25.4)	82 (21.1)	19 (4.9)	175 (45.0)
Maji ya Chai (279)	7 (2.5)	1 (0.4)	6 (2.2)	0 (0.0)	5 (83.3)	1 (16.7)	0 (0.0)
Ngurudoto (282)	12 (4.3)	0 (0.0)	12 (4.3)	7 (58.3)	2 (16.7)	0 (0.0)	3 (25.0)
Bwawani (284)	12 (4.2)	4 (1.4)	8 (2.8)	4 (50.0)	3 (37.5)	0 (0.0)	1 (12.5)
Themi ya Simba (279)	16 (5.7)	0 (0.0)	16 (5.7)	13 (81.3)	1 (6.3)	0 (0.0)	2 (12.5)
ARUSHA (1124)	47 (4.2)	5 (0.4)	42 (3.7)	24 (57.1)	11 (26.2)	1 (2.4)	6 (14.3)
TOTAL (3489)	915 (26.2)	128 (3.7)	787 (22.6)	164 (20.8)	209 (26.6)	38 (4.8)	376 (47.8)

^a^% of selected isolates; *qPCR* Quantitative polymerase chain reaction; *RDT* Rapid diagnostic tests; *LM* Light microscopy; *hrp* histidine rich protein

### Participant selection and collection of blood samples

Blood samples were collected from individuals aged one year and older residing in selected villages (Supplementary Table S1). A team of researchers visited participants’ households, obtained informed consent, and screened individuals for asymptomatic *P. falciparum* infection. Asymptomatic individuals were defined as those who were afebrile, body temperature of 37 ℃ or below, had not experienced any malaria symptoms in the preceding 4–5 days, and had not taken antimalarial medication in the past 7 days. Household selection was based on lists provided by village administrative offices, and participants were enrolled using a randomized sampling approach. To ensure even representation, no more than five eligible individuals were selected per household.

### Sample collection, malaria diagnosis, and DNA extraction

From each participant, venous blood was collected, followed by malaria diagnosis performed using HRP2-based RDT kits (Bioline™ Malaria Ag *P.f/Pan*: Abbott, Chicago, USA) and LM, following standard diagnostic guidelines. For molecular analysis, 50 µl of whole blood was spotted onto Whatman 3MM filter paper to prepare dried blood spots (DBS). The DBS cards were dried overnight, individually sealed in plastic bags with desiccants, and transported at ambient temperature to Kangwon National University (KNU), Republic of Korea. Subsequently, DNA was extracted at KNU from a single DBS spot (corresponding to 50 µl of whole blood), using the QIAamp DNA Mini Kits (Qiagen, Hilden, Germany), following the manufacturer’s instructions. DNA was eluted in a final volume of 50 µl.

### Molecular diagnosis of *P. falciparum* infections

To confirm *P. falciparum* mono-infections and assess DNA quality, a quantitative polymerase chain reaction (qPCR) targeting the small subunit of the *18S rRNA* gene was performed using previously published species-specific primers (Forward: ATTGCTTTTGAGAGGTTTTGTTACTTT, Reverse: GCTGTAGTATTCAAACACAATGAACTCAA) and a probe (FAM-CATAACAGACGGGTAGTCAT) [26]. Reactions were carried out in 10 µl volumes containing 5 µl of 2 × Prime Time Gene Expression Master Mix with ROX reference dye (Integrated DNA Technologies, Coralville, USA), 1 µl of gDNA, 5 pmol of each primer and 2.5 pmol of the *P. falciparum*-specific probe. Amplifications were performed on an AriaMx Real-Time PCR system (Agilent, Santa Clara, USA) under the following conditions; initial polymerase activation at 95 ℃ for 3 min, followed by 40 cycles of denaturation at 95 ℃ for 15 s and annealing/extension at 58 ℃ for 1 min. All reactions were conducted in duplicate. Double-distilled water (DDW) was used as a negative control, and 3D7 strain cultures containing 5% ring-stage parasites, as described previously [27], were used as the positive control for both standard curve generation and estimation of parasite density (relative parasitaemia). Only samples confirmed to be *P. falciparum* mono-infections were included in the subsequent analysis of *pfhrp2* and *pfhrp3* gene deletions. To ensure selection of mono-infection cases, samples that tested positive by qPCR but showed non-*falciparum* antigens (*Pan*-positive) by RDT were excluded. The limit of detection (LoD) of the qPCR assay, based on the 3D7 *18S rRNA* gene standard curve, was 5 parasites/µl. Samples below this threshold were considered analytically negative and excluded from *pfhrp2* and *pfhrp3* gene deletion analysis.

For comparative analyses of parasite density and diagnostic performance, parasite densities estimated by qPCR were categorized into five levels: very low (< 100 parasites/µl), low (100–999 parasites/µl), moderate (1000–4999 parasites/µl), high (5000–99,999 parasites/µl), and hyper parasitaemia (≥ 100,000 parasites/µl), following previously established classifications [28].

### Detection of *pfhrp2* and *pfhrp3* gene deletions

**Fig. 1:**
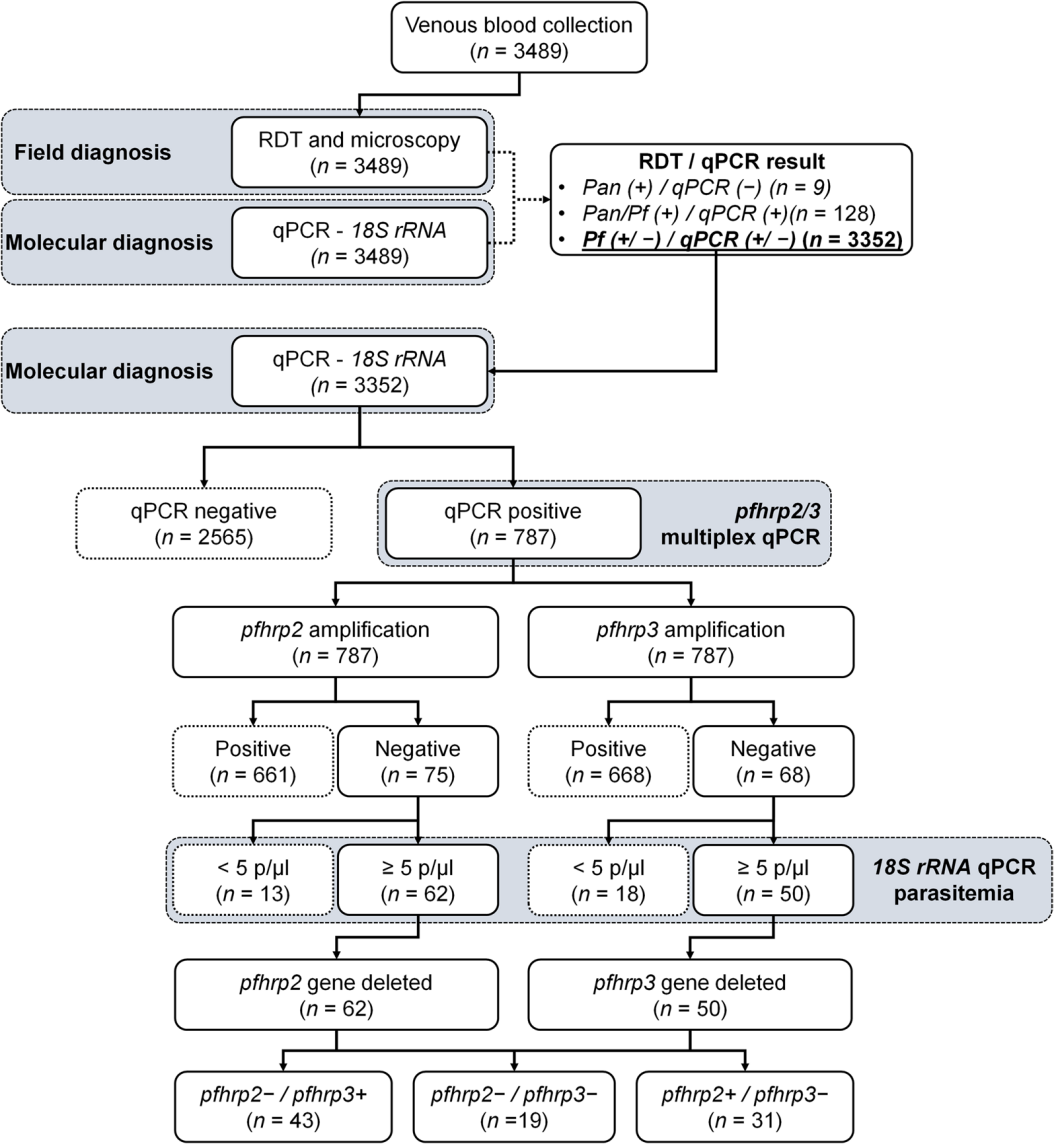
Flowchart of sample processing and determination of *pfhrp2* and *pfhrp3* gene deletions. The diagram illustrates the stepwise analysis of field-collected blood samples, beginning with initial malaria diagnosis using RDTs and LM. Samples then underwent molecular confirmation by qPCR to detect *Plasmodium falciparum* infection. qPCR-positive samples were further analysed using multiplex qPCR to assess the presence or absence of the *pfhrp2* and *pfhrp3* genes. Proportions of samples retained at each stage of processing are shown, culminating in the final distribution of samples with single and dual gene deletions. This flowchart highlights sample attrition across steps and the relative prevalence of gene deletions among confirmed *P. falciparum* infections.* qPCR* Quantitative polymerase chain reaction; *RDT* Rapid diagnostic tests; *LM* Light microscopy; *pfhrp2 Plasmodium falciparum *histidine rich protein 2; *pfhrp3 Plasmodium falciparum *histidine rich protein 3; *p*/*µl* parasites/µl

Multiplex qPCR for the detection of *pfhrp2* and *pfhrp3* gene deletions was confirmed using a laboratory reference strain of *P. falciparum* 3D7 (both *pfhrp2* and *pfhrp3* present), Dd2 (*pfhrp2* deleted and *pfhrp3* present), and HB3 (*pfhrp2* present and *pfhrp3* deleted) [27]. Gene-specific amplification was conducted using previously published primers and probes. For *pfhrp2*, the forward primer was GTATTATCCGCTGCCGTTTTTGCC, and the reverse primer was CATCTACATGTGCTTGATTTTCGT. For *pfhrp3*, the forward was ATATTATCCGCTGCCGTTTTTGCT and the reverse primer was CCTGCATGTGCTTGACTTTCGT. The probes used were FAM-labelled probe (TTCCGCATTTAATAATAACTTGTGTAGC) for *pfhrp2,* and HEX-labelled probe (CTCCGAATTTAACAATAACTTGTTTAGC) for *pfhrp3* [29]. The reaction conditions and volumes used for the multiplex qPCR were identical to those described for the *18S rRNA* qPCR assay. Samples with a relative parasite density below 5 parasites/µl were excluded from gene deletion analysis [29]. For samples suspected to have deletions in *pfhrp2*, *pfhrp3*, or both genes, amplification was repeated in three independent reactions. A gene was considered deleted only if concordant results were obtained in at least two of the three replicates (Fig. 1).

### Statistical analysis

Amplification curves were evaluated using AriaMx 1.8 software (Agilent Technologies), and data visualization was performed using GraphPad Prism 8 (GraphPad Prism Software, San Diego, USA). Parasitaemia was log_10_-transformed, and normality was assessed using the Shapiro-Wilk test. Parasite densities measured by LM and estimated by qPCR were then compared using Pearson’s correlation analysis in SigmaPlot 12 (Systat Software Inc., San Jose, USA). Deletion data were organized in Microsoft Excel (Microsoft, Redmond, USA) using double-entry tables to ensure clarity and minimize entry errors. Unpaired *t*-tests and one-way analysis of variance (ANOVA) were performed to evaluate associations between log_10_-transformed parasitaemia levels and gene deletion profiles.

Diagnostic performance metrics, including sensitivity, specificity, positive predictive value (PPV), negative predictive value (NPV), and accuracy, were calculated using qPCR as the reference standard with the MedCalc’s diagnostic test calculator (https://www.medcalc.org/calc/diagnostic_test.php). The differences in specificity and sensitivity between RDT and LM were statistically analysed using the McNemar test. All statistical tests were conducted at a 95% confidence interval (*CI*) and *P* < 0.05 was considered statistically significant.

## Results

### Study population

A total of 3489 participants were enrolled in the study, including 2365 (67.8%) from high-transmission villages and 1124 (32.2%) from low-endemicity villages. All participants were tested in the field using HRP2-based RDTs and LM. *P. falciparum* mono-infections were subsequently confirmed using species-specific qPCR and RDT results. Among participants from high-transmission regions, 1220 (51.6%) were from Geita and 1145 (48.4%) were from Kigoma (Table 1).

### Field diagnostic performance of RDTs and LM

**Fig. 2:**
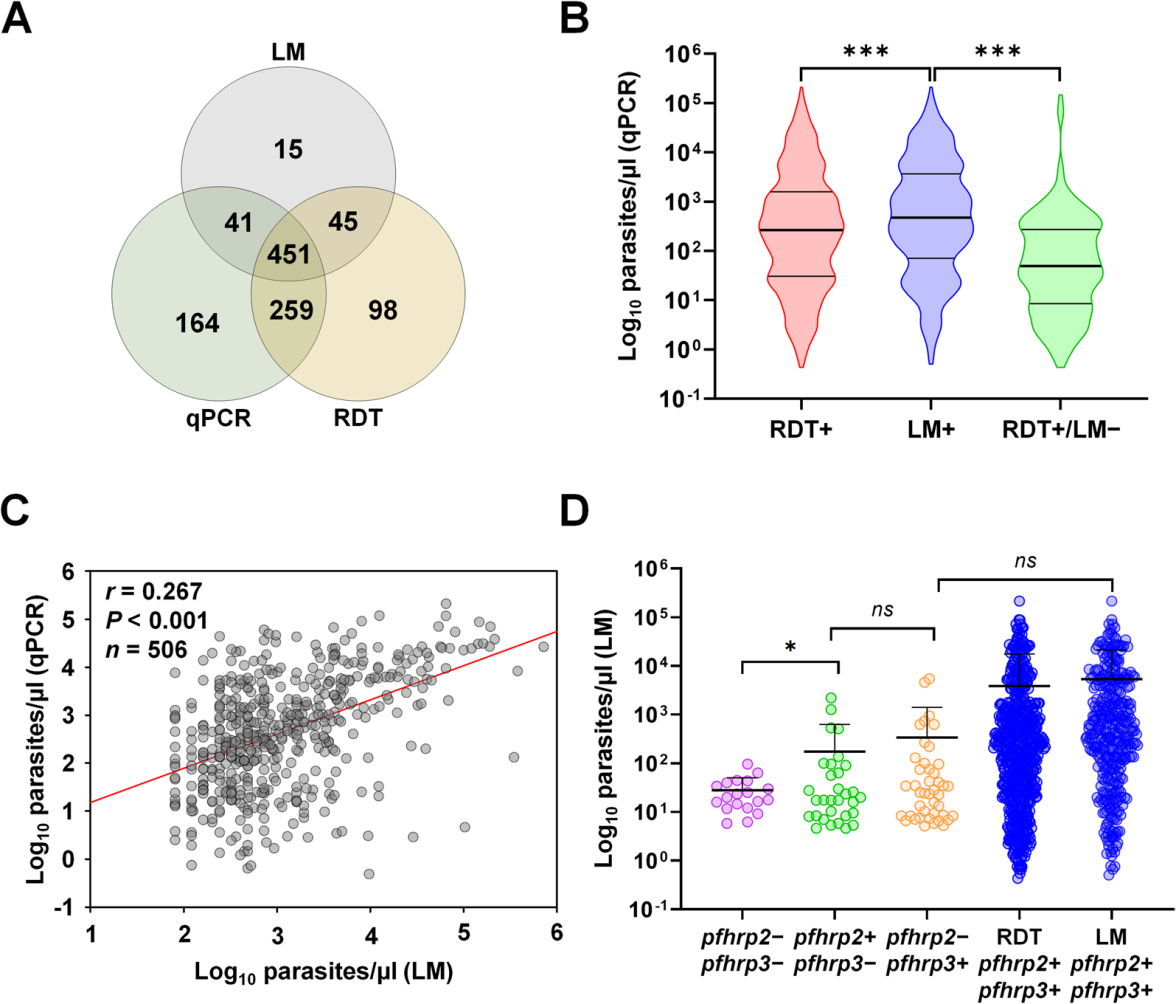
Detection of *Plasmodium falciparum* infections across diagnostic assays and analysis of parasite densities. **A** Venn diagram showing the overlap in *P. falciparum* detection among three diagnostic assays: LM, RDT, and qPCR. A majority of samples (*n* = 451) were positive by all three methods, followed by those detected by both RDT and qPCR. Among samples detected by a single method, qPCR identified the highest number of infections (*n* = 164), exceeding those detected by RDT or LM alone. **B** Comparison of relative parasite densities (as determined by qPCR) between diagnostic outcomes. Parasite densities are shown as log_10_ parasites/μl, and normality of the transformed data was assessed using the Shapiro-Wilk test. Samples positive by both RDT and LM showed higher relative parasitaemia (Unpaired *t*-test, *P* > 0.05), whereas samples missed by LM had significantly lower parasite densities (*P* < 0.001). **C** Correlation analysis between parasitaemia estimated by LM and relative parasite density measured by qPCR (log_10_-transformed). Pearson correlation was used to assess the association (*r* = 0.267, *P* < 0.001). **D** Comparison of relative parasite densities among isolates with different *pfhrp2* and *pfhrp3* deletion statuses (log_10_-transformed). Mean parasite densities were 5534 ± 17,479 parasites/μl for *pfhrp2* + */pfhrp3* + isolates, 2580 ± 9184 for *pfhrp2* − */pfhrp3* + , 5347 ± 16,305 for *pfhrp2* + */pfhrp3* − , and 1654 ± 3654 for *pfhrp2* − */pfhrp3* − isolates. No statistically significant differences were observed among groups (One-way ANOVA, *P* > 0.05). *qPCR* Quantitative polymerase chain reaction; *RDT* Rapid diagnostic tests;* LM* Light microscopy;* pfhrp2 Plasmodium falciparum *histidine rich protein 2; *pfhrp3 Plasmodium falciparum* histidine rich protein 3

To evaluate the performance of HRP2-based RDTs and LM in detecting asymptomatic *P. falciparum* infections, their sensitivity, specificity, and accuracy were assessed using qPCR as the reference standard. Both mono- and co-infections with *P. falciparum* were included in the analysis. Nine out of 3489 samples (0.3%) showed *Pan*-positive results by RDT but were negative by *P. falciparum*-specific qPCR, suggesting mono-infections with non-*falciparum* species (Fig. 1). These samples were excluded from further analysis. Detection rates of asymptomatic *P. falciparum* varied among the three diagnostic methods. A total of 451 (12.9%) participants tested positive by all three methods. RDTs detected 853 (24.4%) participants as positive, whereas LM detected 552 (15.8%) (Fig. 2A). Overall, qPCR identified *P. falciparum* infections in 915 (26.2%) participants, including 128 (3.7%) that were RDT *Pan/Pf* double-positive (Fig. 1 and Table 1).

Among the 915 qPCR-confirmed positive cases, the RDT detected 710 [sensitivity: 77.6% (95% *CI* 74.8–80.3)], whereas LM identified only 492 [sensitivity: 53.8% (95% *CI* 50.5–57.0)]. The RDT also produced more false positives (143 cases, 5.6%) than LM (60 cases, 2.3%), while LM missed more infections (423 cases, 46.2%) compared with the RDT (205 cases, 22.4%) (Table 2). Overall accuracy was slightly higher for RDTs [90.0% (95% *CI* 89.0–91.0)] than for LM [86.2% (95% *CI* 85.0–87.3)]. A McNemar test confirmed that the difference in sensitivity between RDTs and LM was statistically significant (*P* < 0.001). In terms of specificity, LM showed slightly higher values [97.7% (95% *CI* 97.0–98.2)] than RDTs [94.4% (95% *CI* 93.5–95.3)], and this difference was also statistically significant (*P* < 0.001). PPV was higher for LM [89.1% (95% *CI* 86.4–91.4)] than RDTs [83.2% (95% *CI* 80.8–85.4)], whereas the NPV was higher for RDTs [92.2% (95% *CI* 91.3–93.1)] than for LM [85.6% (95% *CI* 84.7–86.4)]. Overall, RDTs exhibited "substantial" agreement with qPCR [κ = 0.736 (95% *CI* 0.710–0.763)], while LM demonstrated only "moderate" agreement [κ = 0.590 (95% *CI* 0.556–0.624)] (Table 2).

**Table 2:** Diagnostic accuracy of microscopy and RDT using qPCR as reference standard

	RDT	Microscopy
TP (qPCR = 915)	710	492
FP (qPCR negative)	143	60
TN (qPCR = 2574)	2431	2514
FN (qPCR positive)	205	423
Sensitivity % (95% *CI*)	77.6 (74.8–80.3)	53.8 (50.5–57.0)
Specificity % (95% *CI*)	94.4 (93.5–95.3)	97.7 (97.0–98.2)
PPV % (95% *CI*)	83.2 (80.8–85.4)	89.1 (86.4.8–91.4)
NPV % (95% *CI*)	92.2 (91.3–93.1)	85.6 (84.7–86.4)
Accuracy %	90.0 (89.0–91.0)	86.2 (85.0–87.3)
kappa value (95% *CI*)	0.736 (0.710–0.763)	0.590 (0.556–0.624)

*qPCR* Quantitative polymerase chain reaction; *RDT* Rapid diagnostic tests; *TP* True positive; *FP* False positive; *TN* True negative; *FN* False negative; *PPV* Positive predictive value; *NPV* Negative predictive value

### Parasite densities and diagnostic concordance

Analysis of diagnostic performance according to parasite density showed that both RDT and LM were more effective in detecting infections with higher parasite densities. However, the detection rate of RDT was significantly higher than that of LM (*P* < 0.001). Furthermore, RDTs identified a significantly greater number of infections with low parasite densities compared to LM (*P* < 0.001) (Fig. 2B). As expected, the sensitivity of both RDT and LM increased in parallel with rising parasite densities. Notably, RDT consistently demonstrated higher sensitivity than LM across all parasitaemia categories (Table 3). A comparison between parasite densities measured by LM and relative parasitaemia estimated by qPCR revealed a significant positive correlation (*r* = 0.267, *P* < 0.001), indicating a weak but statistically meaningful relationship between the two quantification methods (Fig. 2C).

**Table 3:** Sensitivity of RDTs and microscopy across different parasite densities

Parasite density	No. of cases	Sensitivity, *n* (%)	
Parasites/μl of blood	qPCR	LM	RDT
Very low (< 100)	371	124 (33.4)	238 (63.2)
Low (100–999)	283	165 (58.3)	239 (84.5)
Moderate (1000–4999)	127	92 (72.4)	109 (85.8)
High (5000–99,999)	129	106 (82.2)	119 (92.2)
Hyper parasitemia (≥ 100,000)	5	5 (100.0)	5 (100.0)
Total	915	492 (53.2)	710 (76.8)

*qPCR* Quantitative polymerase chain reaction; *RDT* Rapid diagnostic tests; *LM* Light microscopy

### Prevalence and distribution of *pfhrp2* and *pfhrp3* gene deletions

Only isolates confirmed as *P. falciparum* positive were analysed for *pfhrp2* and *pfhrp3* gene deletions. Of the 3489 samples, 915 (26.2%) were positive by qPCR. Among these, 128 (3.7%) were also *Pan*-positive by RDT, indicating possible mixed-species infections. These samples were excluded from further multiplex qPCR analysis for *pfhrp2/3* deletions, leaving 787 (22.6%) isolates confirmed as *P. falciparum* mono-infections for *pfhrp2/3* gene deletion analysis (Fig. 1 and Table 1). Of the 787 isolates selected for deletion analysis, 376 (47.8%) were positive by all three diagnostic methods (RDT, LM, and qPCR), while 164 (20.8%) were positive only by qPCR (Table 1).

Multiplex qPCR identified at least one gene deletion (*pfhrp2* or *pfhrp3*) in 93/787 (11.8%) isolates, including 76 (9.7%) from high-transmission villages and 17 (2.2%) from low-transmission villages. The deletions were unevenly distributed across study sites (Table 4). The single *pfhrp2* deletions were detected in 43 (5.5%) isolates, with a mean relative parasitaemia of 101.0 parasites/μl (range: 5.6–25,106). Single *pfhrp3* deletions were observed in 31 (3.9%) isolates, with a mean parasitaemia of 177.5 parasites/μl (range: 6.6–3214). Dual *pfhrp2/3* deletions were found in 19 (2.4%) isolates, with a significantly lower mean parasitaemia of 27.8 parasites/μl (range: 5.8–96.1). No *pfhrp2/3* dual deletions were detected in Maji ya Chai and Ngurudoto (both located in Arusha District, Meru Region), or Rwantaba (Chato District) (Table 4).

**Table 4:** Prevalence and distribution of *pfhrp2* and *pfhrp3* gene deletions in studied sites and across different demographic parameters

Region	District	Village (*n* of selected isolates)	*pfhrp2* deletion (*n*, %^a^)	*pfhrp3* deletion (*n*, %^a^)	Dual deletion (*n*, %^a^)
Geita	Nyang’hwale	Nyangalamila (94)	6 (6.4)	4 (4.3)	2 (2.1)
		Kayenze (77)	2 (2.6)	1 (1.3)	2 (2.6)
	Chato	Rwantaba (86)	3 (3.5)	1 (1.2)	0 (0.0)
		Ihanga (99)	6 (6.1)	3 (3.0)	1 (1.0)
Kigoma	Kibondo	Kumuhasha (73)	2 (2.7)	3 (4.1)	4 (5.4)
		Bunyambo (100)	9 (9.0)	6 (6.0)	2 (2.0)
	Kasulu	Nyamnyusi (115)	5 (4.3)	3 (2.6)	3 (2.6)
		Mugombe (101)	1 (1.0)	4 (4.0)	3 (3.0)
Total in high transmission (*n* = 745)			34 (4.6)	25 (3.4)	17 (2.3)
Arusha	Meru	Maji ya Chai (6)	2 (33.3)	0 (0.0)	0 (0.0)
		Ngurudoto (12)	3 (25.0)	2 (1.7)	0 (0.0)
	Arusha DC	Bwawani (8)	1 (12.5)	3 (37.5)	1 (12.5)
		Themi ya Simba (16)	3 (18.8)	1 (6.3)	1 (6.3)
Total in low transmission (*n* = 42)			9 (21.4)	6 (14.3)	2 (4.8)
Total in studied regions (*n* = 787)			43 (5.5)	31 (3.9)	19 (2.4)

^a^% of analyzed isolates; *pfhrp2* *Plasmodium falciparum* histidine rich protein 2; *pfhrp3* *Plasmodium falciparum* histidine rich protein 3

Of the 93 isolates with *pfhrp2/3* gene deletions, 53 (57.0%) were from female participants, while the remaining 40 (43.0%) were from males. Proportions of deletions were consistently higher among females across all deletion types, though gender-based differences were not statistically significant. When stratified by age, children under 15 years showed a higher prevalence of overall deletions at 59 (63.4%) cases, compared to participants aged 16 and above, who accounted for 34 (36.6%) of gene deletion cases. This age-related difference was statistically significant (*P* < 0.01), suggesting a higher deletion prevalence in younger individuals (Table 5).

**Table 5:** Comparison of *pfhrp2/3* gene deletions by gender and age of participants

	*pfhrp2* deletion (*n* = 43, %^a^)	*pfhrp3* deletion (*n* = 31, %^a^)	Dual deletion (*n* = 19, %^a^)	*P*-value
Gender				
Males	19 (44.2)	13 (41.9)	8 (42.1)	*P* > 0.05
Females	24 (55.8)	18 (58.1)	11 (57.9)	
Age in years				
< 5	16 (37.2)	8 (25.8)	3 (15.8)	*P* < 0.05
5–15	17 (39.5)	10 (32.3)	5 (26.3)	
16–40	6 (14.0)	9 (29.0)	4 (21.1)	
> 40	4 (9.3)	4 (12.9)	7 (36.8)	

^a^% of analyzed isolates;  *pfhrp2 Plasmodium falciparum *histidine rich protein 2; *pfhrp3 Plasmodium falciparum* histidine rich protein 3

### Impact of gene deletions on diagnostic outcomes

Overall, parasites with gene deletions had lower parasitaemia than those without deletions. Notably, isolates with dual deletions exhibited significantly lower parasitaemia compared with non-deleted samples (*P* < 0.05), while no significant difference was observed for single deletions (Fig. 2D). All dual-deletion isolates were undetected by RDTs, yielding false-negative results compared with qPCR. The impact of *pfhrp2* and *pfhrp3* gene deletions on diagnostic outcomes was evaluated by comparing the proportion of deletion-carrying isolates detected by RDT and LM. Among 43 isolates with a *pfhrp2* − */pfhrp3* + genotype, 24 (55.8%) were detected by RDT and 16 (37.2%) by LM (Table 6). In 31 isolates with a *pfhrp2* + */pfhrp3* − genotype, 27 (87.1%) were RDT positive and 12 (38.7%) were detected by LM (Table 6). Of the 19 isolates with dual *pfhrp2/pfhrp3* deletions, 15 (78.9%) were detected only by LM (Table 6).

**Table 6:** Positivity rates of samples with *pfhrp2* and *pfhrp3* deletions by RDT and microscopy

qPCR (*pfhrp2/3* gene deletion)	RDT (*n*, %^a^)		Microscope (*n*, %^a^)	
	Positive	Negative	Positive	Negative
*pfhrp2* − */pfhrp3* + (*n* = 43)	24 (55.8)	19 (44.2)	16 (37.2)	27 (62.8)
*pfhrp2* + */pfhrp3* − (*n* = 31)	27 (87.1)	4 (12.9)	12 (38.7)	19 (61.3)
*pfhrp2* − */pfhrp3* − (*n* = 19)	0 (0.00)	19 (100)	15 (78.9)	4 (21.1)

^a^% of analyzed isolates

*qPC*R Quantitative polymerase chain reaction; *RDT* Rapid diagnostic tests; *pfhrp2 Plasmodium falciparum* histidine rich protein 2; *pfhrp3 Plasmodium falciparum* histidine rich protein 3

## Discussion

Elimination of malaria in SSA countries is challenged by several factors including the increase in asymptomatic malaria and parasites carrying gene deletions in the *pfhrp2* and *pfhrp3* genes. Here, we present data from asymptomatic malaria patients sampled from twelve villages with varying malaria endemicity in Tanzania, including eight villages within high malaria transmission areas and four villages within low transmission areas [25]. Although *pfhrp2* and *pfhrp3* deletions have been documented in symptomatic patients, the present study specifically examines their prevalence among asymptomatic carriers in Tanzania [30, 31]. To ensure reliability of results, samples that failed to amplify in the multiplex qPCR were independently re-tested. Results were considered valid only if at least three replicates yielded concordant results [30].

As suggested by WHO, the performance of malaria diagnostic tools should be frequently assessed, as lowered parasitaemia, particularly in asymptomatic cases, is expected to reduce their efficiency [32, 33]. The present study revealed that LM missed almost 46.2% of infections, whereas RDT missed nearly 22.4% of qPCR-positive *P. falciparum* infections. These findings are consistent with previous studies on clinical samples, which also showed that LM missed more cases than RDT [34–36]. Overall, the results indicate that RDTs are more sensitive than LM in detecting asymptomatic infections with low parasite densities. False negatives are a significant concern, especially when associated with asymptomatic cases, as failure to detect and treat these individuals allows for continued silent transmission [36]. Although false-negatives in HRP2-based RDTs can result from gene deletions, a large proportion of the current RDT false-negative results (17.5%) were due to other factors, while only 2.6% were attributed to dual *pfhrp2* and *pfhrp3* gene deletions. Previous studies have shown that submicroscopic malaria infections, characterized by low parasitaemia, largely account for the high false-negative rates of LM and RDT [37, 38]. Similarly, in the present study, a comparison of relative parasitaemia in samples detected by RDT and LM showed that many infections with fewer than 100 parasites/µl were detected by RDT but missed by LM, contributing to its lower sensitivity.

Interestingly, the positivity rates for *P. falciparum* infections were slightly lower for RDT (853 cases, 24.4%) than for qPCR (915 cases, 26.2%). Among the RDT positives, 143 cases (4.1% of all samples) were false positives. This may be due to the persistence of malaria antigens in the blood even after the parasites have been cleared [35, 39]. The presence of other infections or non-*falciparum* parasites that can cross-react with RDT may also contribute to the false-positive results [39]. Conversely, 205 RDT-negative cases were positive by qPCR, resulting in a total of 348 (10.0%) discordant cases. McNemar’s test confirmed that the difference in individual-level positivity between RDT and qPCR was highly significant (*P* < 0.001), suggesting that reliance on RDTs alone may underestimate true infection prevalence, particularly in low-density or asymptomatic infections. In this study, the RDT demonstrated higher sensitivity than LM, detecting a greater proportion of asymptomatic *P. falciparum* infections and showing clear advantage in identifying true infections. Although the PPV of the RDT (83.2%) was slightly lower than that of LM (89.1%), the higher NPV (92.2%) indicates strong reliability in ruling out infection. The overall accuracy of RDT (90.0%) exceeded that of LM (86.2%), and the higher kappa value (0.736 vs. 0.590) reflects more consistent performance across all cases. Collectively, these findings demonstrate that while both methods are generally reliable, RDTs provide a more sensitive and consistent tool for detecting asymptomatic *P. falciparum* infections, particularly in field settings where accurate identification of both positive and negative cases is critical. These findings are consistent with other studies conducted elsewhere [35, 36]. The combination of RDT and the molecular qPCR in this study provides evidence that most asymptomatic *P. falciparum* infections in Tanzania produce sufficient HRP2/3 antigen levels to yield positive results on HRP2-based RDTs.

While the gender-based difference in *pfhrp2/3* gene deletion prevalence was not statistically significant, the consistently higher rates observed among females may be influenced by both behavioural and biological factors. In many malaria endemic settings, women, particularly mothers and caregivers, tend to seek health care for themselves and their children more frequently, increasing the likelihood of parasite detection and treatment, which can drive the selection of deletion carrying strains [40]. Additionally, although less studied, hormonal and immunological differences may influence susceptibility and parasite dynamics in females [41]. More notably, our study found a significantly higher prevalence of *pfhrp2/3* deletions among children under 15 years of age. This finding aligns with previous research indicating that younger children, having not yet developed sufficient immunity, are more susceptible to higher-density infections and may be more likely to harbour deletion-carrying parasites [42]. In regions where HRP2-based RDTs are heavily relied upon, especially for febrile children, selective pressure may favour the survival and transmission of HRP2-negative strains, contributing to their increased prevalence in both women and young children.

Although the overall prevalence of *pfhrp2/3* gene deletions was low (11.8%), a key finding was that both single and dual deletions are circulating among asymptomatic *P. falciparum* infections in Tanzania. Regions with higher malaria transmission had a greater proportion of deletions (9.7%) than those within low transmission (2.2%). This study aligns with findings from a study conducted in the northern part of Tanzania, where *pfhrp2/3* deletions were also observed among asymptomatic malaria patients [19]. In contrast to the previous study, which found no *pfhrp2/3* dual deletions, the present study identified dual *pfhrp2/3* gene deletions in 2.4% of isolates, all of which showed discordance between RDT and qPCR results [19, 20]. The prevalence of *pfhrp2/3* gene deletions, particularly in asymptomatic individuals, highlights the importance of surveying and documenting cases across different stages of malaria infection to understand their role in transmission. In this analysis, Pan-only RDT-positive samples were excluded to minimize the risk of misclassification from mixed-species infections. Nevertheless, it is possible that some of these cases represented *P. falciparum* with *pfhrp2* deletions, which would lead to a modest underestimation of deletion prevalence in our study. This potential source of bias should be considered when interpreting the findings and indicates the importance of including sensitivity analyses in future surveillance efforts.

These findings challenge the WHO master protocol for surveillance of *pfhrp2/3* deletions, which emphasizes a health center-based approach [43], thereby increasing the likelihood of missing deletions in asymptomatic individuals. We anticipate that continued use of HRP-based RDTs may exert selective pressure on the parasite population, potentially driving the spread of *pfhrp2/3*-deleted strains [44]. Considering the increasing prevalence of *pfhrp2/3* gene deletions, we recommend that Tanzania’s national malaria diagnostic policy incorporate alternative tools, such as pLDH-based RDTs, which are unaffected by these deletions. Although slightly less sensitive than HRP2-based tests [45], pLDH-based diagnostics offer a viable alternative, particularly in high-risk regions. A combined diagnostic approach using both HRP2 and pLDH-based RDTs could serve as an effective interim solution while long-term policy adjustments are developed.

## Conclusions

This study provides important evidence on the performance of HRP2-based RDTs and LM in detecting asymptomatic *P. falciparum* infections in Tanzania, highlighting both their utility and limitations in the context of emerging *pfhrp2/3* gene deletions. While RDTs demonstrated higher sensitivity and better concordance with qPCR than LM, the prevalence of *pfhrp2/3* gene deletion-carrying infections remains a concern. Notably, the detection of single and dual *pfhrp2/3* deletions, especially in high transmission areas, underscores the silent risk posed by these asymptomatic carriers to malaria control efforts. As a cross-sectional study, these findings are limited by the inability to evaluate temporal trends or causality. In addition, the exclusion of RDT-*Pan* only infections from molecular deletion analysis may have underestimated the true prevalence of *pfhrp2/3* deletions. Despite these limitations, the study emphasizes the need for continued molecular and longitudinal surveillance and calls for more inclusive diagnostic strategies to ensure effective malaria control and elimination in Tanzania.

## Supplementary Information

Supplementary Material 1.

## Data Availability

The data that support the findings of this study are available at Kangwon National University, where they were used under the ethical permit for the current study from the National Institute for Medical Research (NIMR) Tanzania. Data is available from the authors upon reasonable request and with permission.

## Abbreviations

FN: False negative
FP: False positive
HRP2-based RDTs: Histidine rich protein 2-based rapid diagnostic tests
KNU: Kangwon National University
pLDH: *Plasmodium* lactate dehydrogenase
LM: Light microscopy
LOD: Limit of detection
NIMR: National Institute for Medical Research
NMCP: National Malaria Control Program
NPV: Negative predictive value
*pfhrp2*: *Plasmodium falciparum histidine rich protein 2*
*pfhrp3*: *Plasmodium falciparum histidine rich protein 3*
PPV: Positive predictive value
qPCR: Quantitative polymerase chain reaction
RDTs: Rapid diagnostic tests
SSA: Sub-Saharan African
TMDA: Tanzania Medical Device Authority
TN: True negative
TP: True positive
WHO: World Health Organization
