# Supplementary material for: Diagnostic challenges of histidine-rich protein 2-based rapid diagnostic tests due to pfhrp2 and pfhrp3 gene deletions in asymptomatic malaria in Tanzania
Source: Infect Dis Poverty. 2026 Mar 9;15:31. doi: 10.1186/s40249-025-01397-3 (PMC12969855; doi:10.1186/s40249-025-01397-3)
Supplement: Supplementary file 1 — Supplementary Material 1. [file 40249_2025_1397_MOESM1_ESM.docx]

**Supplementary File**

**Diagnostic challenges of histidine-rich protein 2-based rapid diagnostic tests due to *pfhrp2* and *pfhrp3* gene deletions in asymptomatic malaria in Tanzania**

**Ernest Mazigo^1,2^, Hojong Jun^1,3^, Wang-Jong Lee^1,3^, Johnsy Mary Louis^1^, Fadhila Fitriana^1^, Jadidan Hada Syahada^1^, Fauzi Muh^3^, Wanjoo Chun^4^, Won Sun Park^5^, Se Jin Lee^6^, Sunghun Na^6^, Eun-Teak Han^1^, Feng Lu^7^, Winifrida Kidima^8^, Jin-Hee Han^1,3,9*^**

^1^ Department of Medical Environmental Biology and Tropical Medicine, School of Medicine, Kangwon National University, Chuncheon, Republic of Korea

^2^ Department of Parasitic Diseases, National Institute for Medical Research, Dar es Salaam, Tanzania

^3^ Department of Epidemiology and Tropical Diseases, Faculty of Public Health, Universitas Diponegoro, Semarang, Indonesia

^4^ Department of Pharmacology, School of Medicine, Kangwon National University, Chuncheon, Republic of Korea

^5^ Department of Physiology, School of Medicine, Kangwon National University, Chuncheon, Republic of Korea

^6^ Department of Obstetrics and Gynecology, Kangwon National University Hospital, Chuncheon, Republic of Korea

^7^ Department of Pathogen Biology and Immunology, School of Medicine, Yangzhou University, Yangzhou, China

^8^ Department of Zoology, College of Natural and Applied Sciences, University of Dar es Salaam, Dar es Salaam, Tanzania

^9^ Institute of Medical Sciences, Kangwon National University, Chuncheon, Republic of Korea

**Corresponding Author:** Jin-Hee Han, Department of Medical Environmental Biology and Tropical Medicine, Kangwon National University School of Medicine, Chuncheon, Gangwon, Republic of Korea., Email: [han.han@kangwon.ac.kr](mailto:han.han@kangwon.ac.kr)

**Table S1. Geographic coordinates of survey sites.**

| Transmission  profile | District | Region | Village | Latitude (°S) | Latitude (°E) |
| --- | --- | --- | --- | --- | --- |
| High  transmission | Geita | Chato | Ihanga | S2.96512–S2.97296 | E31.65187–E31.68993 |
|  |  |  | Rwantaba | S2.97102 | E31.66093 |
|  |  | Nyang’hwale | Kayenze | S2.39195 | E33.07735 |
|  |  |  | Nyangalamila | S3.15053–S3.93631 | E32.44501–E32.74337 |
|  | Kigoma | Kasulu | Mugombe | S4.57011 | E30.09565 |
|  |  |  | Nyamnyusi | S4.49828 | E30.24316 |
|  |  | Kibondo | Bunyambo | S3.62799 | E30.60003 |
|  |  |  | Kumuhasha | S3.64914 | E30.83602 |
| Low  transmission | Arusha | Arusha DC | Bwawani | S3.60780 | E36.84264 |
|  |  |  | Themi ya Simba | S3.58097 | E36.78507 |
|  |  | Meru | Maji ya Chai | S3.37041 | E36.89445 |
|  |  |  | Ngurudoto | S3.29187 | E36.92708 |
